# Efficacy of Dietary Supplements in Inflammatory Bowel Disease and Related Autoimmune Diseases

**DOI:** 10.3390/nu12072156

**Published:** 2020-07-20

**Authors:** Priyanka Jadhav, Yan Jiang, Karolin Jarr, Cosima Layton, Judith F. Ashouri, Sidhartha R. Sinha

**Affiliations:** 1 Northwestern University, Evanston, IL 60208, USA; priyankajadhav2021@u.northwestern.edu; 2 Division of Gastroenterology & Hepatology, Department of Medicine, Stanford University, Redwood City, CA 94063, USA; yjiang24@stanford.edu (Y.J.); jarr@stanford.edu (K.J.); 3 University of North Carolina at Chapel Hill, Chapel Hill, NC 27599, USA; cosi@live.unc.edu; 4 Rosalind Russell and Ephraim P. Engleman Rheumatology Research Center, Division of Rheumatology, Department of Medicine, University of California, San Francisco, CA 94143, USA

**Keywords:** inflammatory bowel disease, psoriasis, rheumatoid arthritis, prebiotics, probiotics, inflammation, ulcerative colitis, Crohn’s disease, UC, CD

## Abstract

The microbiome is an important contributor to a variety of fundamental aspects of human health, including host metabolism, infection, and the immune response. Gut dysbiosis has been identified as a contributor to the errant immune response in a variety of immune-mediated inflammatory diseases (IMIDs), such as inflammatory bowel disease (IBD), rheumatoid arthritis (RA), and psoriatic disease (psoriasis and psoriatic arthritis). Given this, probiotics and prebiotics have been investigated as therapeutic options in these disease states. In our review, we highlight the current evidence on prebiotics and probiotics as well as other supplements (such as fish oils, vitamin D, and curcumin) as therapies for IBD. Recommendations, however, regarding the specific use of such supplements in IBD have been lacking, particularly from professional societies, often due to study limitations related to small sample sizes and design heterogeneity. Hence, we additionally examine the literature on the use of prebiotics, probiotics, and other supplements in related IMIDs, namely RA and psoriasis/psoriatic arthritis, as these diseases share many approved therapeutic options with IBD. Based on these combined findings, we offer additional evidence that may help guide clinicians in their treatment of patients with IBD (and other IMIDs) and provide recommendations on potential next steps in therapeutic research in this area.

## 1. Introduction

The microbiome is a key contributor to various fundamental aspects of human health, including host metabolism, infection, and the immune response [1,2]. It is composed of 1000–1500 species of bacteria as well as fungi and viruses, whose diversity is important for the maintenance of the metabolic system and maturation of intestinal immunity [3]. An imbalance of these gut microbial communities has been shown to disrupt host immunity and has been linked to intestinal disease [4]. While we are only beginning to understand the complexity of interactions between the microbiome and the host immune system, it has been shown that gut dysbiosis is one of the key contributors to errant host immune responses in a variety of immune-mediated inflammatory diseases (IMIDs) [5,6,7,8]. As such, there is strong rationale to suspect demonstrable therapeutic potential with prebiotic and probiotic usage in inflammatory bowel disease (IBD), a chronic relapsing intestinal disease without medical cure, and other IMIDs. With growing supporting evidence, the interest in probiotics and prebiotics, as well as additional supplements, to modulate disease has dramatically grown [9,10]. While the importance of supplements to correct certain aspects of IBD, such as mineral deficiencies [11,12], is well-established, the role of other dietary supplements in affecting the overall disease course is less clear.

The incidence of IBD—including both Crohn’s disease (CD), which can affect any part of the gastrointestinal tract, and ulcerative colitis (UC), which is restricted to the colon—is increasing worldwide [13]. Similar to IBD, there appears to be a dynamic and tightly regulated crosstalk between dysbiosis-associated microbes and the gut-associated immune response seen prior to the onset of related autoimmune diseases, such as rheumatoid arthritis (RA), psoriasis, and psoriatic arthritis [14]. In fact, more recent observations implicate a causative role for dysbiosis in the development of inflammatory arthritides [14,15,16]. RA, psoriatic disease (psoriasis and psoriatic arthritis), and IBD also share several treatments [17,18,19], which highlight some common final pathways in the pathogenesis of these heterogeneous diseases. In addition, it appears that several common dietary interventions can result in disease modifications across these IMIDs [14,20].

Recent literature increasingly supports the use of prebiotics, probiotics, and other supplements to treat gut dysbiosis [14,21]. Despite this, however, there are few clear guidelines advocating for their use as a treatment for IBD, and results in clinical trials have generally been equivocal. In fact, the American Gastroenterology Association (AGA) practice guidelines on the role of probiotics in management of gastrointestinal disorders do not recommend their use outside of a clinical trial as a result of knowledge gaps caused by low sample sizes as well as study design and treatment heterogeneity [21]. However, there is still much to extract and extrapolate from these IBD studies. Similarly, there are several well-designed studies investigating supplements in other IMIDs, namely RA and psoriasis, that offer opportunities to better ascertain their potential in mitigating disease. In this review, we examine the data surrounding probiotics, prebiotics, and other dietary supplements not only in IBD but also in RA and psoriatic disease. Through this cross-analysis, we hope to better understand which forms of supplementation have been most effective, and which provide the most potential for future therapeutic use.

## 2. Materials and Methods

We performed a focused literature review using the PubMed database to identify relevant articles related to IMIDs and dietary supplements. We searched using lexicon commonly used to describe the IMID (e.g., ulcerative colitis, UC, Crohn’s disease, Crohn’s, rheumatoid arthritis, RA, etc.) combined with terms to describe the supplement of interest (e.g., prebiotic, probiotic, vitamin D, etc.). We reviewed a variety of types of research articles, including systematic reviews and meta-analyses, randomized controlled trials, and observational studies. We generally limited our data to human studies; however, in areas where clinical research was limited, basic science research pertaining to the proposed mechanism of action or in vitro effects were also included. Though not a systematic review, studies were excluded if they were deemed lower quality (very small sample sizes, poor methodology) by a consensus among our research team, which included gastroenterology and rheumatology physicians.

## 3. Results

### 3.1. Probiotics

#### 3.1.1. Use in Inflammatory Bowel Disease

Mounting evidence suggests that the complex interplay between genetics, environmental factors, immune defenses, and the gut microbiome promotes the development of IBD [22,23,24]. This has generated interest in how probiotics may be harnessed to mediate the mucosal inflammation of IBD [22]. Different strains of probiotics have shown promise in the treatment of both active disease and the maintenance of disease remission in IBD [25,26,27,28,29,30,31].

In active IBD, there are additional efficacy differences found in UC and CD. Though there have been numerous studies investigating probiotic usage and outcomes in UC, the diversity of probiotic usage and variance in study design have limited the conclusions that can be drawn from meta-analyses [31,32]. This being said, there have been studies showing the therapeutic benefit of probiotics either alone or in conjunction with standard-of-care therapy in UC [26,27]. Specifically, there are several trials that support the use of VSL#3—a multi-strain probiotic mixture—to induce remission in patients with mild-to-moderate UC [27]. A double-blind randomized study investigating standard pharmaceutical treatment plus VSL#3 supplementation versus placebo for 8 weeks found a significant reduction in the ulcerative colitis disease activity index (UCDAI, which assesses stool frequency, rectal bleeding, endoscopic mucosal appearance, and physician’s rating of disease activity) (*p* = 0.01) and rectal bleeding (*p* = 0.014) [33]. Improvements in stool frequency, physician’s rate of disease activity, and endoscopic scores were also seen, but these did not reach statistical significance. These findings support VSL#3 as a potential add-on to conventional pharmacologic therapy [33]. In contrast to VSL#3, the probiotic bacterium *Escherichia coli* Nissle 1917 (EcN) does not seem to improve disease and may, in fact, worsen clinical outcomes in active IBD. EcN has been tested in a double-blind randomized study for 8 weeks as an adjunctive therapy to ciprofloxacin in mild-to-moderate UC. Though there were no significant differences found in the ciprofloxacin/placebo versus ciprofloxacin/EcN groups, patients in the EcN/placebo group had poorer outcomes. In fact, fewer patients reached remission (*p* < 0.05) in the EcN/placebo treatment group compared to placebo/placebo, and experienced greater withdrawals (*p* < 0.05) compared to other groups combined [34].

Published literature on probiotic use as therapy for active CD is more limited. The available study results are difficult to generalize due to their non-randomized trial methodology or small patient populations [35]. Further investigation is required to make generalizable prognostic statements about probiotic use in the induction of disease remission for IBD, but the evidence supporting their use in UC is more promising than in CD. Perhaps ongoing studies evaluating the efficacy of probiotic adjunctive therapies in UC—including one investigating the effects of *Lactobacillus rhamnosus* GG [36] and another one investigating SER-287 (microbiome therapy containing a consortium of live bacterial spores) [37] on mild-to-moderate UC—will further elucidate the effects of probiotics on clinical disease outcomes.

Similar to treatment of active disease in IBD, the evidence of probiotic usage for the maintenance of remission in both UC and CD has also been limited and mixed. Despite EcN not showing efficacy in active UC, there is evidence supporting its use in maintaining remission. In a 12-month double-blind study, there was no clinically significant difference in maintenance of remission between patients who took the probiotic monotherapy twice a day versus mesalamine, a standard therapy for mild-to-moderate UC, three times a day (*p* < 0.01 for equivalence) [28]. This suggests that probiotics may provide an alternative to conventional therapies to maintain disease remission. Furthermore, a meta-analysis conducted by Sang et al. analyzed 13 studies and found that probiotic treatment was more effective than placebo for maintaining remission in UC [29]. However, prior to initiating all UC patients on probiotics, clear guidance should be provided as there appear to be species and strain efficacy differences. In a recent Japanese study, for example, investigating *Bifidobacterium + Lactobacillus* treatment versus placebo in quiescent UC, the study was terminated early after the 48-week follow-up due to a lack of efficacy as no significant difference was found between the two arms in relapse-free survival (*p* = 0.64) [38]. These mixed results may also reflect differences in genetics and environmental exposures, in addition to highlighting differences based on probiotic composition. Thus, though studies show promise for probiotic use in disease as maintenance therapy, additional well-controlled trials are necessary to understand the impact of various probiotics on clinical disease outcomes.

In CD, there are more data investigating probiotic use as a maintenance therapy (compared to its use in active CD). Though some studies have suggested that probiotics may have efficacy in maintaining CD remission [39], this has not been consistently reflected in improved clinical markers of disease activity [40,41,42]. In a study of VSL#3 in preventing the relapse of CD after surgery, inflammatory cytokines were found to be significantly reduced between the VSL#3 versus placebo groups (*p* < 0.05). A trend for improvement in the number of endoscopic lesions and severe recurrence with early VSL#3 treatment (*p* = 0.09) was also observed. However, no differences in clinical and disease activity index scores were found between the two groups [30]. An additional study investigating the effect of *Saccharomyces boulardii* on time to relapse in patients with CD showed no significant differences between the probiotic and placebo groups (*p* = 0.78) [42].

#### 3.1.2. Use in RA and Psoriasis

While the majority of probiotic research in IMIDs has been conducted in IBD, there are several studies investigating their efficacy in patients with RA and psoriasis (Table 1). Evaluating the efficacy of probiotics in patients with RA and psoriasis, in addition to IBD, may provide a generalized framework to understand its utility in the treatment of autoimmune inflammatory diseases, and provide further insights into its role in the treatment of IBD.

Trials investigating the effects of probiotic use in patients with RA have also shown clinical and symptomatic improvement. In two randomized double-blind clinical trials, there was a significant reduction in serum proinflammatory cytokine levels found in patients receiving *Lactobacillus casei* [43,44]. Two trials also found a significant reduction in C-reactive protein (CRP) and Disease Activity Score (DAS-28, consisting of tender and swollen joint count, CRP, and visual analog scale of global health) after treatment with *Lactobacillus casei* [44,53]. A randomized double-blind trial investigating the effect of probiotic *Bacillus coagulans GBI-30* also demonstrated improvements in patient pain global assessment (Patient Pain Assessment *p* = 0.05, Pain Scale *p* < 0.05) and with improvement, albeit limited, in self-diagnosed disability (ability to walk *p* = 0.07, ability to reach *p* = 0.11) [45]. The promising efficacy of these probiotics in RA suggests they may be altering, and potentially addressing, the contribution of dysbiosis to disease activity. If so, this could provide an additional framework for its use in patients with IBD, or at least for those suffering from IBD-associated arthropathy.

Similar to RA, some studies exist assessing probiotic use and clinical outcomes in patients with psoriasis. From these limited studies, it appears that probiotic mixtures including strains of *Lactobacillus* and *Bifidobacterium* have some efficacy on disease activity. One randomized double-blind study investigating a combination probiotic mixture of *Bifidobacterium longum, B. lactis,* and *Lactobacillus rhamnosus* showed a reduction of the Psoriasis Area and Severity Index (PASI) up to 75% (*p* < 0.05), and an improvement in the Physician Global Assessment Index (48.9% in the probiotic group received 0 or 1, representative of remission, compared to 30.2% in the placebo group) [46]. Additionally, a study done on only the effect of *Lactobacillus pentosus* in a mouse model of psoriasis demonstrated a significant reduction in scaling lesions and the gene expression of proinflammatory cytokines [54], providing additional evidence for the beneficial use of *Lactobacillus* species in the treatment of psoriasis. Though both *Lactobacillus* and *Bifidobacterium* strains have indicated potential in treating psoriasis and IBD, there is evidence that the inflammatory pathways targeted by these probiotics varies by disease. One study investigating *Bifidobacterium infantis* 35624 across IBD, psoriasis, and chronic fatigue syndrome (CFS) showed that it induced a reduction in CRP across all three conditions. The effects, however, on different proinflammatory markers between IBD versus CFS and psoriasis differed. Tumor necrosis factor alpha (TNF-α) was reduced in psoriasis (*p* = 0.04) and CFS (*p* = 0.02), while interleukin (IL)-6, for example, was reduced in CFS (*p* = 0.05) and UC (*p* = 0.06) [55]. The differing cytokine responses could, at least in part, also be explained by the different background therapies the patients with CFS, psoriasis or IBD were on. Overall, the efficacy of a variety of *Lactobacillus* strains in both RA as well as psoriasis, in addition to some therapeutic benefit seen in IBD, indicate that these strains are likely reasonable candidates to consider for therapeutic use.

### 3.2. Prebiotics

Diet has a known impact on maintaining intestinal microbial homeostasis. Prebiotics are food products that promote the growth of microorganisms thought to benefit the host [9,48,56]. We commonly think of prebiotics as non-digestible fibers and nutrients that promote a healthier gut microbiome through the stimulation of growth of healthy bacteria. Types of prebiotics, including fructans (polymers of fructose molecules) and galacto-oligosaccharides (a type of oligosaccharide that is not hydrolyzed by humans but rather fermented by gut bacteria), can be found naturally in foods, such as asparagus, beets, garlic, and lentils to name a few [56]. Compared to probiotic usage, trials investigating the clinical efficacy of prebiotics are substantially more limited. Even so, studies in IBD reveal beneficial clinical effects of prebiotics, specifically fiber and oligosaccharides.

Variations of inulin, common soluble fibers such as those found in chicory root, have shown promise. One study investigating the effects of inulin on clinical symptoms of patients with UC (Mayo score 3–8) found a significant reduction in the high-treatment group (*p* = 0.04) associated with a significant increase in colonic butyrate production (*p* = 0.04), a short-chain fatty acid with anti-inflammatory effects [48]. This offers a proposed mechanism of effect, as butyrate production inversely correlates with clinical symptoms [57]. Other studies found that oligofructose-enriched inulin significantly decreased the Rachmilewitz score [58], an index used to assess endoscopic disease activity (*p* < 0.05), as well as the fecal inflammatory marker calprotectin (*p* < 0.05) in patients with mild-to-moderate UC after 14 days [59]. Additionally, germinated barley foodstuff was found to significantly reduce CRP levels (*p* = 0.02), as well as abdominal pain and cramping (*p* = 0.02) compared to placebo in mild-to-moderate patients with UC [60]. It also appeared to improve other clinical signs of IBD (e.g., episodes of diarrhea, visible blood in stool) compared to baseline, though these did not meet statistical significance [60].

Interestingly, prebiotic use in CD not only reduces disease activity [61,62] but may also alter susceptibility to disease. The latter was highlighted in a prospective study, which analyzed data from over 170,000 women, followed over 26 years, who participated in the Nurses’ Health Study to investigate the effects of long-term dietary fiber intake on the risk of developing IBD. Dietary information was obtained via a validated semiquantitative food questionnaire that was administered every 4 years during the follow-up period. Intake of a high-fiber diet, particularly of fruit, was associated with a 40% reduction in CD development (multivariate HR for CD, 0.59; 95% confidence interval (CI), 0.39–0.90) [63]. Prebiotics have also been shown to reduce disease activity in CD. Analysis of prebiotic complex carbohydrates, such as fructans (also known as fructooligosaccharides (FOS)), have shown some promise for patients with CD. A small study (*n* = 10) demonstrated FOS induced remission, as defined by a reduction in a commonly used clinical disease activity measure, the Harvey Bradshaw Index (HBI), from 9.8 to 6.9 (*p* < 0.01) [61]. A larger study (*n* = 103) in patients with moderate and severe CD (as defined by the Crohn’s disease activity index, CDAI, ≥220) showed an improvement in CDAI scores compared to placebo, though this did not meet statistical significance (*p* = 0.07) [62].

While there are a paucity of data available on prebiotic use and their clinical effects on the inflammatory arthropathies, there are some data indicating dietary fiber’s ability to restore microbial diversity in patients with RA, likely driven by an increase in the observed circulatory T regulatory cells and a favorable shift in the T helper immune response (Table 1) [47]. Based on these results, and what we know about the positive effects of prebiotics on maintaining intestinal microbial homeostasis [9,48], prebiotics have potential to modulate IBD disease activity. In the future, it will be important to offer additional personalization of prebiotic regimens, perhaps starting with which types are most effective for specific IMIDs.

### 3.3. Other Dietary Supplementation

Non-probiotic/-prebiotic dietary supplements are also commonly used in the treatment of IMIDs [64]. As further described below, several supplements have shown promise in modulating the clinical symptoms associated with IMIDs [65,66,67,68]. Hence, even potentially more than with prebiotics and probiotics, IBD providers, by discerning data from their use in other IMIDs, may be able to identify potential therapeutic supplements for patients with IBD.

#### 3.3.1. Omega-3 Supplementation

Omega-3 (or n-3) fatty acids from sources, such as fish oils, have been used to treat chronic inflammatory disorders, such as IBD [68]. A meta-analysis looking at a total of 1039 patients across 6 studies found that n-3 supplementation may lead to improvement in the maintenance of remission in CD (RR 0.77, 95% CI 0.61 to 0.98) [69]. However, when using evidence from two of the six large high-quality studies, which appeared to be free of bias, the authors concluded that n-3 supplements are probably ineffective for the maintenance of remission in CD [69]. In UC, the effectiveness of n-3 fatty acid oral supplementation was examined alongside other supplements, including FOS, vitamin E, vitamin C, and selenium, in patients with mild-to-moderate disease activity. This combination therapy appeared to reduce the prednisone dose required to alleviate clinical symptoms (*p* < 0.001) [70]. However, attributing the effect to a specific supplement is not possible.

When examining the published literature on the effect of n-3 supplements in other IMIDs, such as RA, psoriasis, and psoriatic arthritis, the results are generally positive. For example, in RA, it is thought that fish oil and omega-3 supplementation may directly inhibit the production of proinflammatory cytokines [71]. In another study of newly diagnosed patients with RA investigating the adjunctive effects of high- versus low-dose fish oil in conjunction with disease-modifying antirheumatic drugs (DMARDs), higher doses were found to have lower DMARD failure (*p* < 0.01) and higher rates of remission (*p* = 0.04) [49]. In psoriasis, the results are more conflicted [72]. In a recent meta-analysis of randomized controlled trials, fish oil did not demonstrate a significant reduction in the severity of psoriasis, and individual trials reviewed over the last decade also yielded mixed results [73]. However, in one of the larger and more well-controlled trials investigating the efficacy of n-3 supplementation versus olive oil in psoriatic arthritis, an improvement was shown in the disease activity score, 68 tender joint count, and PASI scores [50]. There was a significant reduction in the amount of non-steroidal medication necessary to alleviate clinical symptoms in patients receiving n-3 supplementation (*p* = 0.04). Given the favorable outcomes observed in patients with RA and psoriatic arthritis, it is possible that the effects of supplementation may be more evident in patients with inflammatory arthritides. Therefore, it may be worthwhile to explore the use of n-3 supplementation in patients with IBD-associated inflammatory arthritis [74,75]

#### 3.3.2. Vitamin D Supplementation

There has been considerable evidence associating low vitamin D levels and inflammation [76]. Thus, vitamin D supplementation has been studied for possible therapeutic benefit in chronic inflammatory conditions. In a retrospective longitudinal study investigating the relationship between serum vitamin D levels, inflammatory markers, and clinical disease activity in patients with IBD, it was shown that low vitamin D levels correlated with higher fecal calprotectin in UC and CD (*p* < 0.001) and higher CRP levels in UC (*p* = 0.01) [77]. Furthermore, a deficiency in vitamin D_3_ correlated with an increase in disease flares, hospitalizations, and steroid treatment (*p* < 0.01). When examining the literature on other related IMIDs, limited data exist. In a short-term study looking at patients with RA, it appeared that high weekly doses of vitamin D_3_ on a background of methotrexate therapy resulted in a non-significant improvement in clinical disease activity by DAS-28 [51]. Although topical vitamin D is an established treatment in psoriasis [78], its role as an oral therapeutic is less clear. A randomized double-blind trial found that oral vitamin D_3_ supplementation resulted in only a slight improvement in PASI (45% vs. 38%) [79]. Given the known role of vitamin D in maintaining mucosal barriers and the function of T cells [80], further exploration is required to better understand the possible therapeutic potential of vitamin D in patients with inflammatory diseases. Though there are data linking vitamin D deficiency to worse clinical outcomes in patients with IBD, an increased therapeutic vitamin D target level is yet to be determined, which may better guide vitamin D replacement strategies.

#### 3.3.3. Curcumin Supplementation

Curcumin—a strong antioxidant and predominant polyphenol in the turmeric root—has been used in many cultures for millennia for its antioxidative and anti-inflammatory properties [81]. Due to its potential in modulating inflammation, curcumin is commercially offered as an over-the-counter treatment [82]. Though controlled trials directly examining curcumin’s anti-inflammatory effects are limited in IBD, there are some favorable data for its use as an adjunctive anti-inflammatory therapy. In fact, high-dose curcumin (3 g/day) significantly improved disease activity and induced clinical and endoscopic remission in patients with mild-to-moderate UC disease activity on a background of mesalamine therapy [83]. A larger trial with 300 patients with mild-to-moderate UC did not find a significant clinical benefit when using much lower doses of curcumin (450 mg/day) [84]. Larger, randomized controlled trials in RA using moderate doses of curcumin (from 120 mg to 1.2 g a day) demonstrated an improvement in clinical disease activity scores, tender joint count, and swollen joint count [52,85,86]. Though clinical trials of curcumin use in psoriatic disease are limited, in vitro studies suggest a potential benefit [87]. Additionally, topical curcumin treatment demonstrated inhibition of the inflammatory IL-23/IL-17 axis (pathogenic drivers of psoriatic disease) and other inflammatory cytokines (*p* < 0.05) in a mouse model of psoriasis [88]. It appears that curcumin’s anti-inflammatory properties may in fact provide benefit as an adjunctive therapy in inflammatory diseases, though studies suggest perhaps high doses are required to see clinical benefit.

## 4. Discussion

While there are substantial data supporting probiotics, prebiotics, and other dietary supplementation for the treatment of IMIDs, larger better controlled trials need to be performed in order to further determine the efficacy of each of these treatments on specific patient populations. Specific professional society guideline recommendations on their clinical use have generally been lacking, largely due to the heterogeneity of the evidence base. Much, however, can still be extrapolated from these studies to guide providers for management and researchers for promising avenues to further explore [21].

There are several possible mechanisms of action by which dietary supplements may have a therapeutic effect on IBD, including inhibition of microbial pathogens, modification of intestinal permeability, modulation of the immune response of intestinal epithelia and mucosal immune cells, and decomposition of luminal pathogenic antigens [89]. Probiotics, for example, attached to the mucosal surface receptors, can inhibit adhesion and cell invasion by pathogenic bacteria [90,91]. Furthermore, probiotics have been shown to ferment undigested dietary fibers, producing short-chain fatty acids and other acid products that may inhibit the growth of pathogens [92,93]. Short-chain fatty acids themselves have anti-inflammatory properties [94,95]. Probiotics have also been shown to downregulate the expression of proinflammatory cytokines, such as TNFα, IL1β, and interferon gamma by several pathways, including the inhibition of NF-κB (nuclear factor kappa-light-chain-enhancer of activated B cells), modulation of toll like receptor (TLR)-2 signaling, and the PPARγ (peroxisome proliferator activated receptor gamma) pathway [89]. Despite these mechanistic insights, it is still unclear which probiotic strains have the most therapeutic potential in treatment of UC and CD. This further supports the need to conduct cross-analysis and investigation in related IMIDs.

Cocktail strains, such as VSL#3, have the most data supporting adjunctive treatment in IBD, with some conflicting data on *Bifidobacterium* and *Lactobacillus* strains as being potentially useful. The efficacy of *Lactobacillus casei* in RA, as well as of *Lactobacillus* and *Bifidobacterium* strains in psoriasis, gives additional supporting evidence and more basis for providers to potentially utilize these strains for patients with IBD.

Studies assessing the efficacy of prebiotic usage in IBD are more limited, with some data supporting the use of inulin-type fructans for UC and potentially the use of dietary fiber and fructooligosaccharides to prevent CD. There are very limited studies detailing the use of prebiotics in other IMIDs. Currently, given the limited amount of information on prebiotic usage, there is definitely a need for additional testing to be conducted to assess if it can become a viable adjunctive treatment option. However, given prior studies suggesting reasonable mechanisms, such as increased anti-inflammatory byproduct (e.g., butyrate) production in the colon and restoration of microbial diversity, there is solid ground for additional research.

In regard to other supplements, it appears that vitamin D deficiency is linked to disease pathogenesis in IBD, RA, and psoriatic disease. Vitamin D regulates gut mucosal immunity by various means, including the modification of gut epithelial integrity and alteration of T cell development and function [80,96]. With generally widespread availability of vitamin D testing, various target levels should be explored in a broad assortment of IMIDs. Omega-3 supplementation has been shown to inhibit inflammatory cytokine production [97] and to be clinically effective in RA and psoriasis, but this potential therapy needs further assessment before generalizing to IBD treatment. However, given the positive results in other IMIDs, particularly RA, future studies may focus on outcomes of omega-3 supplementation in IBD-associated inflammatory arthropathies [75,76]. Curcumin has a medicinal history of at least 2500 years in Asia and has long been known to have antibacterial properties [81]. There is evidence of the anti-inflammatory and antioxidant effects of curcumin, including downregulation of proinflammatory interleukins and cytokines, and inhibition of inflammatory cells. Curcumin has also been shown to be effective in randomized controlled trials in mild-to-moderate UC and RA, as well as in mouse models of psoriasis. Overall, the positive effects of curcumin in IMIDs provide further support for its use in IBD. In IBD studies, outcomes also differed based on the dose of curcumin given [83,84], so future additional analyses on dose-dependent changes may be fruitful.

There is strong evidence that the gut microbiota play a crucial role in IBD pathogenesis as well as in the development of other IMIDs [5,6,7,8]. Although conflicting results and a lack of comparability of available studies often preclude firm guidance, the available supporting data hold promise. Further research should not only focus on additional investigation of specific bacterial strains but also combinations of strains and combinations of prebiotics and probiotics, as well as interactions of bacteria with fungi and viruses.

## Figures and Tables

**Table 1:** Highlighted studies on dietary supplements in immune-mediated inflammatory diseases.

**Probiotics:** Outcomes related to the use of probiotics in patients with certain IMIDs provide additional guidance for its potential use in patients with IBD.				
**Study**	**Disease**	**Design**	***N***	**Results**
Alipour [43]	RA	RCT	46	Those who received 8-week supplementation with *L casei* had decreased inflammatory markers and disease activity scores.
Zamani [44]	RA	RCT	60	8-week probiotic containing 3 different strains decreased disease activity scores and CRP.
Mandel [45]	RA	RCT	45	2-month supplementation with bacillus strain probiotic improved pain assessment scores.
Navarro-Lopez [46]	Ps *	RCT	90	Higher prevalence of reduced psoriasis area and severity index of up to 75% in those who took 3 months of probiotics.
Tursi [33]	UC	RCT	144	8-week treatment course of VSL#3 decreased disease activity scores and clinical symptoms.
Petersen [34]	UC	RCT	100	Adjunctive therapy with EcN may worsen clinical outcomes compared to placebo.
**Prebiotics:** Limited data exist in both IBD and other IMID populations. There have been a few studies demonstrating proposed mechanism of microbiome and prebiotic interactions, which could be the foundation for additional research.				
**Study**	**Disease**	**Design**	***N***	**Results**
Häger [47]	RA	Prospective cohort	36	High-fiber supplementation for 1 month increased circulating T regulatory cells and decreased markers of bone erosion.
Valcheva [48]	UC	Pilot intervention study	25	15g/day of inulin-type fructans increased production of colonic butyrate, an anti-inflammatory short-chain fatty acid.
**Supplements: Omega-3 supplementation seems promising for potential IBD-related arthropathies.** Curcumin also appears to have a potential role based on positive studies in the IMID population. Further studies on vitamin D and targetable levels are needed.				
**Study**	**Disease**	**Design**	***N***	**Results**
Proudman [49]	RA	RCT	122	Those who received fish oil supplementation had higher rates of remission and reduced DMARD failure rates.
Kristensen [50]	PsA *	RCT	145	The n-3-supplemented group showed improved outcomes in measures for disease activity and a reduction in use of NSAIDs and paracetamol.
Salesi [51]	RA	RCT	117	In those on methotrexate, there was a non-significant difference in efficacy outcomes after 12 weeks of vitamin D compared to placebo.
Amalraj [52]	RA	RCT	36	Improved disease activity scores in those who received both low and high doses of curcumin for 90 days.

Not all studies mentioned in text are included in Table 1. * Ps—psoriasis; PsA—psoriatic arthritis; RCT—randomized, controlled trial; *N*—number of study participants.
